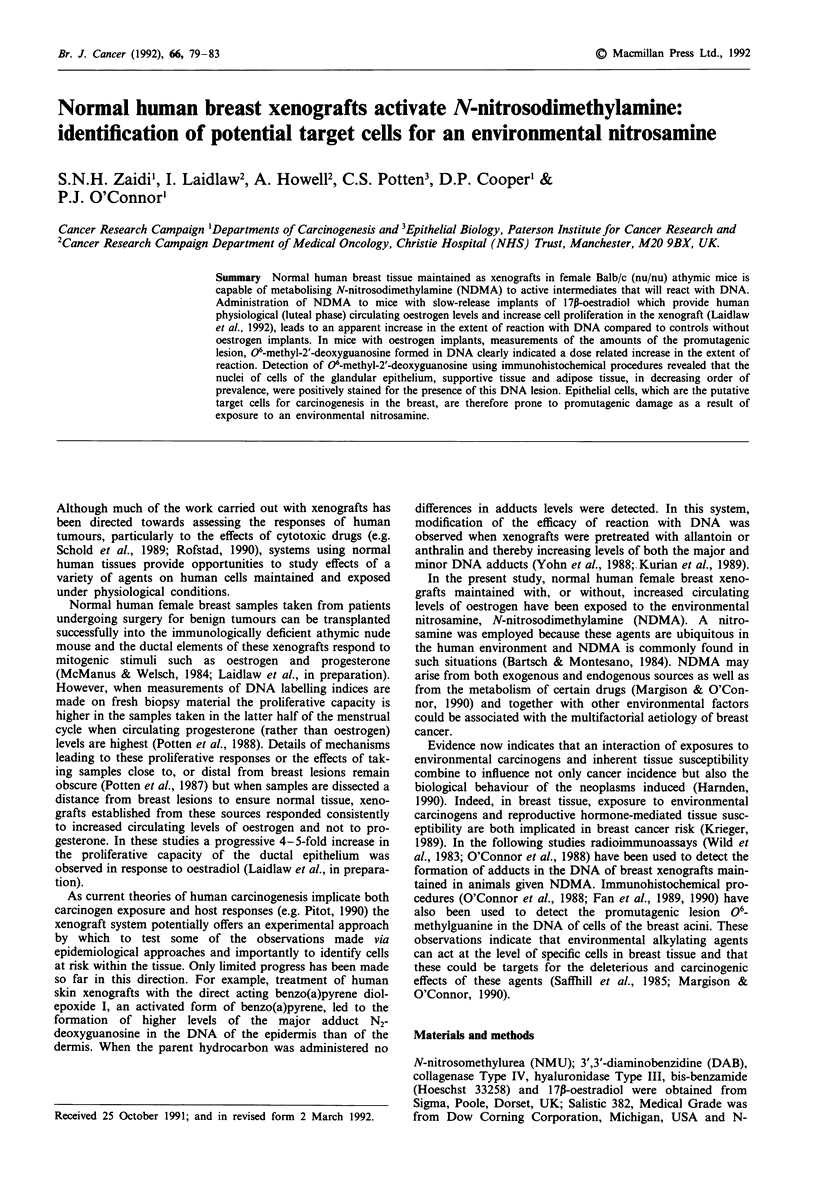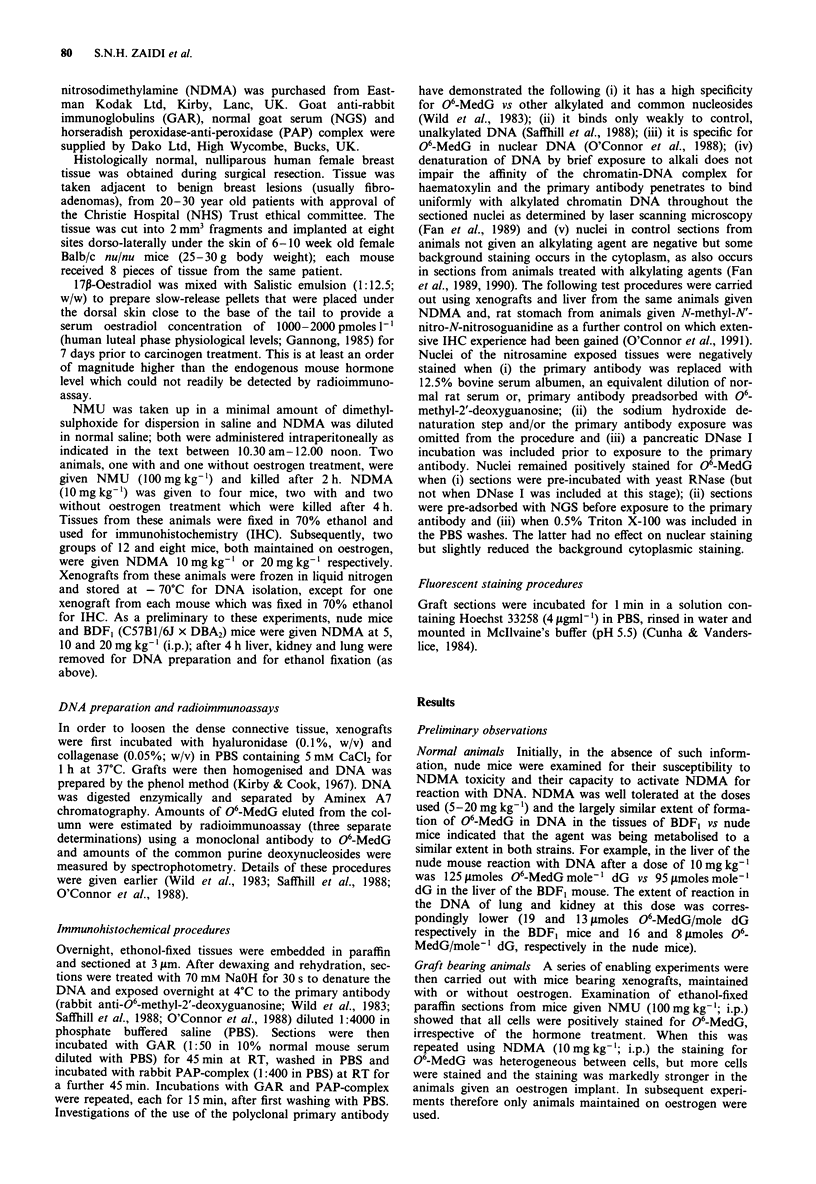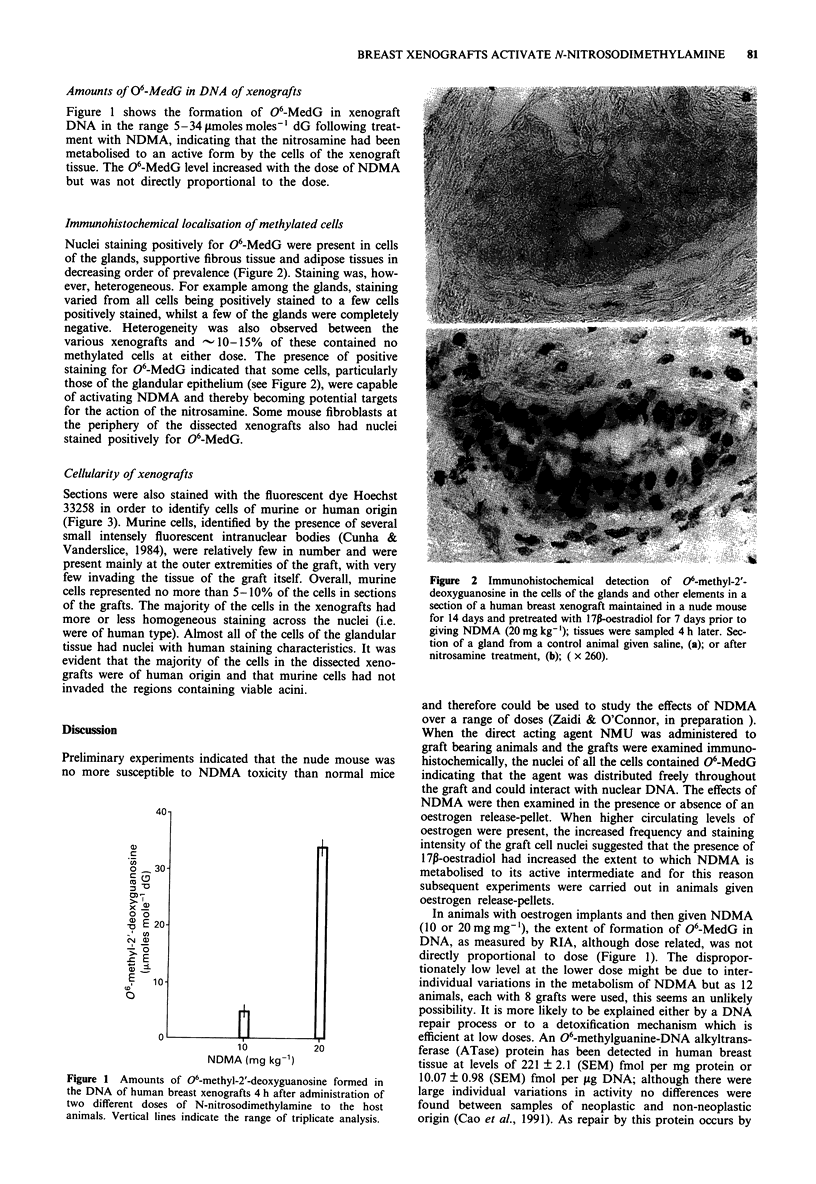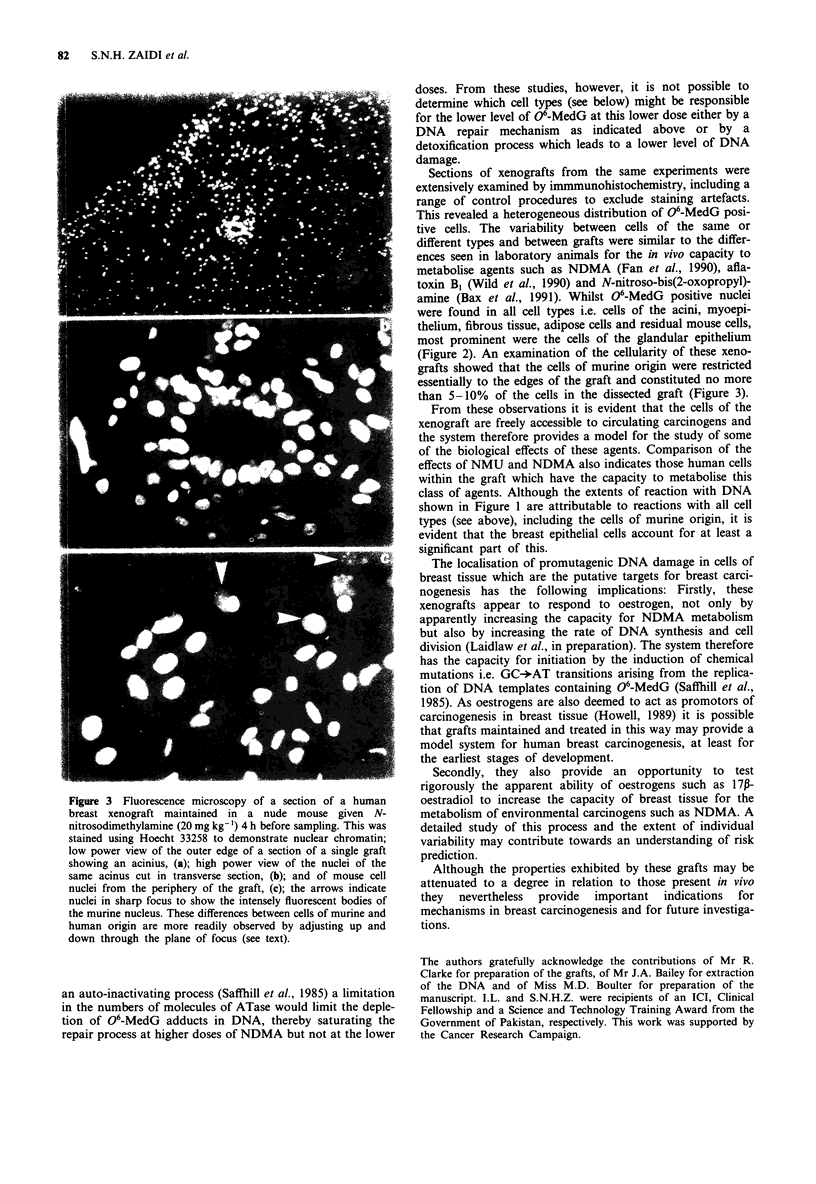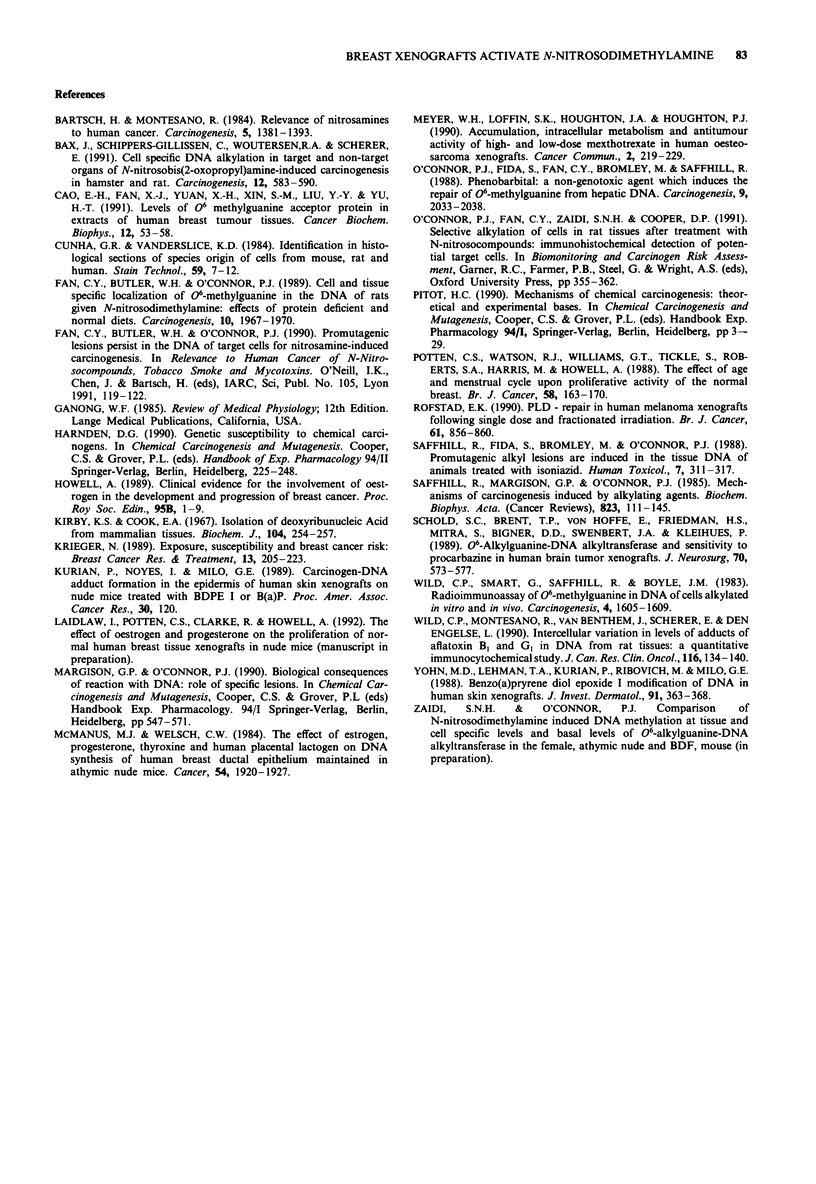# Normal human breast xenografts activate N-nitrosodimethylamine: identification of potential target cells for an environmental nitrosamine.

**DOI:** 10.1038/bjc.1992.220

**Published:** 1992-07

**Authors:** S. N. Zaidi, I. Laidlaw, A. Howell, C. S. Potten, D. P. Cooper, P. J. O'Connor

**Affiliations:** Department of Carcinogenesis, Christie Hospital (NHS) Trust, Manchester, UK.

## Abstract

**Images:**


Br. J. Cancer (1992), 66, 79–83 © Macmillan Press Ltd., 1992

### Normal human breast xenografts activate N-nitrosodimethylamine: identification of potential target cells for an environmental nitrosamine

S.N.H. Zaidi[1], I. Laidlaw[2], A. Howell[2], C.S. Potten[3], D.P. Cooper[1] & P.J. O'Connor[1]

*Cancer Research Campaign [1]Departments of Carcinogenesis and [3]Epithelial Biology, Paterson Institute for Cancer Research and [2]Cancer Research Campaign Department of Medical Oncology, Christie Hospital (NHS) Trust, Manchester, M20 9BX, UK.*

**Summary** Normal human breast tissue maintained as xenografts in female Balb/c (nu/nu) athymic mice is capable of metabolising *N*-nitrosodimethylamine (NDMA) to active intermediates that will react with DNA. Administration of NDMA to mice with slow-release implants of 17β-oestradiol which provide human physiological (luteal phase) circulating oestrogen levels and increase cell proliferation in the xenograft (Laidlaw *et al.*, 1992), leads to an apparent increase in the extent of reaction with DNA compared to controls without oestrogen implants. In mice with oestrogen implants, measurements of the amounts of the promutagenic lesion, $O^6$-methyl-2'-deoxyguanosine formed in DNA clearly indicated a dose related increase in the extent of reaction. Detection of $O^6$-methyl-2'-deoxyguanosine using immunohistochemical procedures revealed that the nuclei of cells of the glandular epithelium, supportive tissue and adipose tissue, in decreasing order of prevalence, were positively stained for the presence of this DNA lesion. Epithelial cells, which are the putative target cells for carcinogenesis in the breast, are therefore prone to promutagenic damage as a result of exposure to an environmental nitrosamine.

Although much of the work carried out with xenografts has been directed towards assessing the responses of human tumours, particularly to the effects of cytotoxic drugs (e.g. Schold *et al.*, 1989; Rofstad, 1990), systems using normal human tissues provide opportunities to study effects of a variety of agents on human cells maintained and exposed under physiological conditions.

Normal human female breast samples taken from patients undergoing surgery for benign tumours can be transplanted successfully into the immunologically deficient athymic nude mouse and the ductal elements of these xenografts respond to mitogenic stimuli such as oestrogen and progesterone (McManus & Welsch, 1984; Laidlaw *et al.*, in preparation). However, when measurements of DNA labelling indices are made on fresh biopsy material the proliferative capacity is higher in the samples taken in the latter half of the menstrual cycle when circulating progesterone (rather than oestrogen) levels are highest (Potten *et al.*, 1988). Details of mechanisms leading to these proliferative responses or the effects of taking samples close to, or distal from breast lesions remain obscure (Potten *et al.*, 1987) but when samples are dissected a distance from breast lesions to ensure normal tissue, xenografts established from these sources responded consistently to increased circulating levels of oestrogen and not to progesterone. In these studies a progressive 4–5-fold increase in the proliferative capacity of the ductal epithelium was observed in response to oestradiol (Laidlaw *et al.*, in preparation).

As current theories of human carcinogenesis implicate both carcinogen exposure and host responses (e.g. Pitot, 1990) the xenograft system potentially offers an experimental approach by which to test some of the observations made *via* epidemiological approaches and importantly to identify cells at risk within the tissue. Only limited progress has been made so far in this direction. For example, treatment of human skin xenografts with the direct acting benzo(a)pyrene diol-epoxide I, an activated form of benzo(a)pyrene, led to the formation of higher levels of the major adduct $N_2$-deoxyguanosine in the DNA of the epidermis than of the dermis. When the parent hydrocarbon was administered no differences in adducts levels were detected. In this system, modification of the efficacy of reaction with DNA was observed when xenografts were pretreated with allantoin or anthralin and thereby increasing levels of both the major and minor DNA adducts (Yohn *et al.*, 1988; Kurian *et al.*, 1989).

In the present study, normal human female breast xenografts maintained with, or without, increased circulating levels of oestrogen have been exposed to the environmental nitrosamine, *N*-nitrosodimethylamine (NDMA). A nitrosamine was employed because these agents are ubiquitous in the human environment and NDMA is commonly found in such situations (Bartsch & Montesano, 1984). NDMA may arise from both exogenous and endogenous sources as well as from the metabolism of certain drugs (Margison & O'Connor, 1990) and together with other environmental factors could be associated with the multifactorial aetiology of breast cancer.

Evidence now indicates that an interaction of exposures to environmental carcinogens and inherent tissue susceptibility combine to influence not only cancer incidence but also the biological behaviour of the neoplasms induced (Harnden, 1990). Indeed, in breast tissue, exposure to environmental carcinogens and reproductive hormone-mediated tissue susceptibility are both implicated in breast cancer risk (Krieger, 1989). In the following studies radioimmunoassays (Wild *et al.*, 1983; O'Connor *et al.*, 1988) have been used to detect the formation of adducts in the DNA of breast xenografts maintained in animals given NDMA. Immunohistochemical procedures (O'Connor *et al.*, 1988; Fan *et al.*, 1989, 1990) have also been used to detect the promutagenic lesion $O^6$-methylguanine in the DNA of cells of the breast acini. These observations indicate that environmental alkylating agents can act at the level of specific cells in breast tissue and that these could be targets for the deleterious and carcinogenic effects of these agents (Saffhill *et al.*, 1985; Margison & O'Connor, 1990).

### Materials and methods

*N*-nitrosomethylurea (NMU); 3',3'-diaminobenzidine (DAB), collagenase Type IV, hyaluronidase Type III, bis-benzamide (Hoeschst 33258) and 17β-oestradiol were obtained from Sigma, Poole, Dorset, UK; Salistic 382, Medical Grade was from Dow Corning Corporation, Michigan, USA and N-

Received 25 October 1991; and in revised form 2 March 1992.

nitrosodimethylamine (NDMA) was purchased from Eastman Kodak Ltd, Kirby, Lanc, UK. Goat anti-rabbit immunoglobulins (GAR), normal goat serum (NGS) and horseradish peroxidase-anti-peroxidase (PAP) complex were supplied by Dako Ltd, High Wycombe, Bucks, UK.

Histologically normal, nulliparous human female breast tissue was obtained during surgical resection. Tissue was taken adjacent to benign breast lesions (usually fibroadenomas), from 20–30 year old patients with approval of the Christie Hospital (NHS) Trust ethical committee. The tissue was cut into 2 $mm^3$ fragments and implanted at eight sites dorso-laterally under the skin of 6–10 week old female Balb/c *nu/nu* mice (25–30 g body weight); each mouse received 8 pieces of tissue from the same patient.

17β-Oestradiol was mixed with Salistic emulsion (1:12.5; w/w) to prepare slow-release pellets that were placed under the dorsal skin close to the base of the tail to provide a serum oestradiol concentration of 1000–2000 pmoles $l^{-1}$ (human luteal phase physiological levels; Gannong, 1985) for 7 days prior to carcinogen treatment. This is at least an order of magnitude higher than the endogenous mouse hormone level which could not readily be detected by radioimmunoassay.

NMU was taken up in a minimal amount of dimethylsulphoxide for dispersion in saline and NDMA was diluted in normal saline; both were administered intraperitoneally as indicated in the text between 10.30 am–12.00 noon. Two animals, one with and one without oestrogen treatment, were given NMU (100 mg $kg^{-1}$) and killed after 2 h. NDMA (10 mg $kg^{-1}$) was given to four mice, two with and two without oestrogen treatment which were killed after 4 h. Tissues from these animals were fixed in 70% ethanol and used for immunohistochemistry (IHC). Subsequently, two groups of 12 and eight mice, both maintained on oestrogen, were given NDMA 10 mg $kg^{-1}$ or 20 mg $kg^{-1}$ respectively. Xenografts from these animals were frozen in liquid nitrogen and stored at −70°C for DNA isolation, except for one xenograft from each mouse which was fixed in 70% ethanol for IHC. As a preliminary to these experiments, nude mice and $BDF_1$ (C57B1/6J × $DBA_2$) mice were given NDMA at 5, 10 and 20 mg $kg^{-1}$ (i.p.); after 4 h liver, kidney and lung were removed for DNA preparation and for ethanol fixation (as above).

### *DNA preparation and radioimmunoassays*

In order to loosen the dense connective tissue, xenografts were first incubated with hyaluronidase (0.1%, w/v) and collagenase (0.05%; w/v) in PBS containing 5 mM $CaCl_2$ for 1 h at 37°C. Grafts were then homogenised and DNA was prepared by the phenol method (Kirby & Cook, 1967). DNA was digested enzymically and separated by Aminex A7 chromatography. Amounts of $O^6$-MedG eluted from the column were estimated by radioimmunoassay (three separate determinations) using a monoclonal antibody to $O^6$-MedG and amounts of the common purine deoxynucleosides were measured by spectrophotometry. Details of these procedures were given earlier (Wild *et al.*, 1983; Saffhill *et al.*, 1988; O'Connor *et al.*, 1988).

### *Immunohistochemical procedures*

Overnight, ethonol-fixed tissues were embedded in paraffin and sectioned at 3 μm. After dewaxing and rehydration, sections were treated with 70 mM NaOH for 30 s to denature the DNA and exposed overnight at 4°C to the primary antibody (rabbit anti-$O^6$-methyl-2′-deoxyguanosine; Wild *et al.*, 1983; Saffhill *et al.*, 1988; O'Connor *et al.*, 1988) diluted 1:4000 in phosphate buffered saline (PBS). Sections were then incubated with GAR (1:50 in 10% normal mouse serum diluted with PBS) for 45 min at RT, washed in PBS and incubated with rabbit PAP-complex (1:400 in PBS) at RT for a further 45 min. Incubations with GAR and PAP-complex were repeated, each for 15 min, after first washing with PBS. Investigations of the use of the polyclonal primary antibody have demonstrated the following (i) it has a high specificity for $O^6$-MedG *vs* other alkylated and common nucleosides (Wild *et al.*, 1983); (ii) it binds only weakly to control, unalkylated DNA (Saffhill *et al.*, 1988); (iii) it is specific for $O^6$-MedG in nuclear DNA (O'Connor *et al.*, 1988); (iv) denaturation of DNA by brief exposure to alkali does not impair the affinity of the chromatin-DNA complex for haematoxylin and the primary antibody penetrates to bind uniformly with alkylated chromatin DNA throughout the sectioned nuclei as determined by laser scanning microscopy (Fan *et al.*, 1989) and (v) nuclei in control sections from animals not given an alkylating agent are negative but some background staining occurs in the cytoplasm, as also occurs in sections from animals treated with alkylating agents (Fan *et al.*, 1989, 1990). The following test procedures were carried out using xenografts and liver from the same animals given NDMA and, rat stomach from animals given *N*-methyl-*N*′-nitro-*N*-nitrosoguanidine as a further control on which extensive IHC experience had been gained (O'Connor *et al.*, 1991). Nuclei of the nitrosamine exposed tissues were negatively stained when (i) the primary antibody was replaced with 12.5% bovine serum albumen, an equivalent dilution of normal rat serum or, primary antibody preadsorbed with $O^6$-methyl-2′-deoxyguanosine; (ii) the sodium hydroxide denaturation step and/or the primary antibody exposure was omitted from the procedure and (iii) a pancreatic DNase I incubation was included prior to exposure to the primary antibody. Nuclei remained positively stained for $O^6$-MedG when (i) sections were pre-incubated with yeast RNase (but not when DNase I was included at this stage); (ii) sections were pre-adsorbed with NGS before exposure to the primary antibody and (iii) when 0.5% Triton X-100 was included in the PBS washes. The latter had no effect on nuclear staining but slightly reduced the background cytoplasmic staining.

### *Fluorescent staining procedures*

Graft sections were incubated for 1 min in a solution containing Hoechst 33258 (4 μg$ml^{-1}$) in PBS, rinsed in water and mounted in McIlvaine's buffer (pH 5.5) (Cunha & Vanderslice, 1984).

### Results

### *Preliminary observations*

*Normal animals* Initially, in the absence of such information, nude mice were examined for their susceptibility to NDMA toxicity and their capacity to activate NDMA for reaction with DNA. NDMA was well tolerated at the doses used (5–20 mg $kg^{-1}$) and the largely similar extent of formation of $O^6$-MedG in DNA in the tissues of $BDF_1$ *vs* nude mice indicated that the agent was being metabolised to a similar extent in both strains. For example, in the liver of the nude mouse reaction with DNA after a dose of 10 mg $kg^{-1}$ was 125 μmoles $O^6$-MedG $mole^{-1}$ dG *vs* 95 μmoles $mole^{-1}$ dG in the liver of the $BDF_1$ mouse. The extent of reaction in the DNA of lung and kidney at this dose was correspondingly lower (19 and 13 μmoles $O^6$-MedG/mole dG respectively in the $BDF_1$ mice and 16 and 8 μmoles $O^6$-MedG/$mole^{-1}$ dG, respectively in the nude mice).

*Graft bearing animals* A series of enabling experiments were then carried out with mice bearing xenografts, maintained with or without oestrogen. Examination of ethanol-fixed paraffin sections from mice given NMU (100 mg $kg^{-1}$; i.p.) showed that all cells were positively stained for $O^6$-MedG, irrespective of the hormone treatment. When this was repeated using NDMA (10 mg $kg^{-1}$; i.p.) the staining for $O^6$-MedG was heterogeneous between cells, but more cells were stained and the staining was markedly stronger in the animals given an oestrogen implant. In subsequent experiments therefore only animals maintained on oestrogen were used.

*Amounts of* $O^6$*-MedG in DNA of xenografts*

Figure 1 shows the formation of $O^6$-MedG in xenograft DNA in the range 5–34 μmoles $moles^{-1}$ dG following treatment with NDMA, indicating that the nitrosamine had been metabolised to an active form by the cells of the xenograft tissue. The $O^6$-MedG level increased with the dose of NDMA but was not directly proportional to the dose.

*Immunohistochemical localisation of methylated cells*

Nuclei staining positively for $O^6$-MedG were present in cells of the glands, supportive fibrous tissue and adipose tissues in decreasing order of prevalence (Figure 2). Staining was, however, heterogeneous. For example among the glands, staining varied from all cells being positively stained to a few cells positively stained, whilst a few of the glands were completely negative. Heterogeneity was also observed between the various xenografts and ~10–15% of these contained no methylated cells at either dose. The presence of positive staining for $O^6$-MedG indicated that some cells, particularly those of the glandular epithelium (see Figure 2), were capable of activating NDMA and thereby becoming potential targets for the action of the nitrosamine. Some mouse fibroblasts at the periphery of the dissected xenografts also had nuclei stained positively for $O^6$-MedG.

*Cellularity of xenografts*

Sections were also stained with the fluorescent dye Hoechst 33258 in order to identify cells of murine or human origin (Figure 3). Murine cells, identified by the presence of several small intensely fluorescent intranuclear bodies (Cunha & Vanderslice, 1984), were relatively few in number and were present mainly at the outer extremities of the graft, with very few invading the tissue of the graft itself. Overall, murine cells represented no more than 5–10% of the cells in sections of the grafts. The majority of the cells in the xenografts had more or less homogeneous staining across the nuclei (i.e. were of human type). Almost all of the cells of the glandular tissue had nuclei with human staining characteristics. It was evident that the majority of the cells in the dissected xenografts were of human origin and that murine cells had not invaded the regions containing viable acini.

### Discussion

Preliminary experiments indicated that the nude mouse was no more susceptible to NDMA toxicity than normal mice

**Figure 1** Amounts of $O^6$-methyl-2′-deoxyguanosine formed in the DNA of human breast xenografts 4 h after administration of two different doses of N-nitrosodimethylamine to the host animals. Vertical lines indicate the range of triplicate analysis.

**Figure 2** Immunohistochemical detection of $O^6$-methyl-2′-deoxyguanosine in the cells of the glands and other elements in a section of a human breast xenograft maintained in a nude mouse for 14 days and pretreated with 17β-oestradiol for 7 days prior to giving NDMA (20 mg $kg^{-1}$); tissues were sampled 4 h later. Section of a gland from a control animal given saline, (**a**); or after nitrosamine treatment, (**b**); ( × 260).

and therefore could be used to study the effects of NDMA over a range of doses (Zaidi & O'Connor, in preparation ). When the direct acting agent NMU was administered to graft bearing animals and the grafts were examined immunohistochemically, the nuclei of all the cells contained $O^6$-MedG indicating that the agent was distributed freely throughout the graft and could interact with nuclear DNA. The effects of NDMA were then examined in the presence or absence of an oestrogen release-pellet. When higher circulating levels of oestrogen were present, the increased frequency and staining intensity of the graft cell nuclei suggested that the presence of 17β-oestradiol had increased the extent to which NDMA is metabolised to its active intermediate and for this reason subsequent experiments were carried out in animals given oestrogen release-pellets.

In animals with oestrogen implants and then given NDMA (10 or 20 mg $mg^{-1}$), the extent of formation of $O^6$-MedG in DNA, as measured by RIA, although dose related, was not directly proportional to dose (Figure 1). The disproportionately low level at the lower dose might be due to interindividual variations in the metabolism of NDMA but as 12 animals, each with 8 grafts were used, this seems an unlikely possibility. It is more likely to be explained either by a DNA repair process or to a detoxification mechanism which is efficient at low doses. An $O^6$-methylguanine-DNA alkyltransferase (ATase) protein has been detected in human breast tissue at levels of 221 ± 2.1 (SEM) fmol per mg protein or 10.07 ± 0.98 (SEM) fmol per μg DNA; although there were large individual variations in activity no differences were found between samples of neoplastic and non-neoplastic origin (Cao *et al.*, 1991). As repair by this protein occurs by

**Figure 3** Fluorescence microscopy of a section of a human breast xenograft maintained in a nude mouse given *N*-nitrosodimethylamine (20 mg $kg^{-1}$) 4 h before sampling. This was stained using Hoecht 33258 to demonstrate nuclear chromatin; low power view of the outer edge of a section of a single graft showing an acinius, (**a**); high power view of the nuclei of the same acinus cut in transverse section, (**b**); and of mouse cell nuclei from the periphery of the graft, (**c**); the arrows indicate nuclei in sharp focus to show the intensely fluorescent bodies of the murine nucleus. These differences between cells of murine and human origin are more readily observed by adjusting up and down through the plane of focus (see text).

an auto-inactivating process (Saffhill *et al.*, 1985) a limitation in the numbers of molecules of ATase would limit the depletion of $O^6$-MedG adducts in DNA, thereby saturating the repair process at higher doses of NDMA but not at the lower doses. From these studies, however, it is not possible to determine which cell types (see below) might be responsible for the lower level of $O^6$-MedG at this lower dose either by a DNA repair mechanism as indicated above or by a detoxification process which leads to a lower level of DNA damage.

Sections of xenografts from the same experiments were extensively examined by immmunohistochemistry, including a range of control procedures to exclude staining artefacts. This revealed a heterogeneous distribution of $O^6$-MedG positive cells. The variability between cells of the same or different types and between grafts were similar to the differences seen in laboratory animals for the *in vivo* capacity to metabolise agents such as NDMA (Fan *et al.*, 1990), aflatoxin $B_1$ (Wild *et al.*, 1990) and *N*-nitroso-bis(2-oxopropyl)-amine (Bax *et al.*, 1991). Whilst $O^6$-MedG positive nuclei were found in all cell types i.e. cells of the acini, myoepithelium, fibrous tissue, adipose cells and residual mouse cells, most prominent were the cells of the glandular epithelium (Figure 2). An examination of the cellularity of these xenografts showed that the cells of murine origin were restricted essentially to the edges of the graft and constituted no more than 5–10% of the cells in the dissected graft (Figure 3).

From these observations it is evident that the cells of the xenograft are freely accessible to circulating carcinogens and the system therefore provides a model for the study of some of the biological effects of these agents. Comparison of the effects of NMU and NDMA also indicates those human cells within the graft which have the capacity to metabolise this class of agents. Although the extents of reaction with DNA shown in Figure 1 are attributable to reactions with all cell types (see above), including the cells of murine origin, it is evident that the breast epithelial cells account for at least a significant part of this.

The localisation of promutagenic DNA damage in cells of breast tissue which are the putative targets for breast carcinogenesis has the following implications: Firstly, these xenografts appear to respond to oestrogen, not only by apparently increasing the capacity for NDMA metabolism but also by increasing the rate of DNA synthesis and cell division (Laidlaw *et al.*, in preparation). The system therefore has the capacity for initiation by the induction of chemical mutations i.e. GC→AT transitions arising from the replication of DNA templates containing $O^6$-MedG (Saffhill *et al.*, 1985). As oestrogens are also deemed to act as promotors of carcinogenesis in breast tissue (Howell, 1989) it is possible that grafts maintained and treated in this way may provide a model system for human breast carcinogenesis, at least for the earliest stages of development.

Secondly, they also provide an opportunity to test rigorously the apparent ability of oestrogens such as 17β-oestradiol to increase the capacity of breast tissue for the metabolism of environmental carcinogens such as NDMA. A detailed study of this process and the extent of individual variability may contribute towards an understanding of risk prediction.

Although the properties exhibited by these grafts may be attenuated to a degree in relation to those present *in vivo* they nevertheless provide important indications for mechanisms in breast carcinogenesis and for future investigations.

The authors gratefully acknowledge the contributions of Mr R. Clarke for preparation of the grafts, of Mr J.A. Bailey for extraction of the DNA and of Miss M.D. Boulter for preparation of the manuscript. I.L. and S.N.H.Z. were recipients of an ICI, Clinical Fellowship and a Science and Technology Training Award from the Government of Pakistan, respectively. This work was supported by the Cancer Research Campaign.

### References

BARTSCH, H. & MONTESANO, R. (1984). Relevance of nitrosamines to human cancer. *Carcinogenesis*, **5**, 1381-1393.

BAX, J., SCHIPPERS-GILLISSEN, C., WOUTERSEN,R.A. & SCHERER, E. (1991). Cell specific DNA alkylation in target and non-target organs of *N*-nitrosobis(2-oxopropyl)amine-induced carcinogenesis in hamster and rat. *Carcinogenesis*, **12**, 583-590.

CAO, E.-H., FAN, X.-J., YUAN, X.-H., XIN, S.-M., LIU, Y.-Y. & YU, H.-T. (1991). Levels of $O^6$ methylguanine acceptor protein in extracts of human breast tumour tissues. *Cancer Biochem. Biophys.*, **12**, 53-58.

CUNHA, G.R. & VANDERSLICE, K.D. (1984). Identification in histological sections of species origin of cells from mouse, rat and human. *Stain Technol.*, **59**, 7-12.

FAN, C.Y., BUTLER, W.H. & O'CONNOR, P.J. (1989). Cell and tissue specific localization of $O^6$-methylguanine in the DNA of rats given *N*-nitrosodimethylamine: effects of protein deficient and normal diets. *Carcinogenesis*, **10**, 1967-1970.

FAN, C.Y., BUTLER, W.H. & O'CONNOR, P.J. (1990). Promutagenic lesions persist in the DNA of target cells for nitrosamine-induced carcinogenesis. In *Relevance to Human Cancer of N-Nitrosocompounds, Tobacco Smoke and Mycotoxins*. O'Neill, I.K., Chen, J. & Bartsch, H. (eds), IARC, Sci, Publ. No. 105, Lyon 1991, 119-122.

GANONG, W.F. (1985). *Review of Medical Physiology*; 12th Edition. Lange Medical Publications, California, USA.

HARNDEN, D.G. (1990). Genetic susceptibility to chemical carcinogens. In *Chemical Carcinogenesis and Mutagenesis*. Cooper, C.S. & Grover, P.L. (eds). *Handbook of Exp. Pharmacology* 94/II Springer-Verlag, Berlin, Heidelberg, 225-248.

HOWELL, A. (1989). Clinical evidence for the involvement of oestrogen in the development and progression of breast cancer. *Proc. Roy Soc. Edin.*, **95B**, 1-9.

KIRBY, K.S. & COOK, E.A. (1967). Isolation of deoxyribunucleic Acid from mammalian tissues. *Biochem. J.*, **104**, 254-257.

KRIEGER, N. (1989). Exposure, susceptibility and breast cancer risk: *Breast Cancer Res. & Treatment*, **13**, 205-223.

KURIAN, P., NOYES, I. & MILO, G.E. (1989). Carcinogen-DNA adduct formation in the epidermis of human skin xenografts on nude mice treated with BDPE I or B(a)P. *Proc. Amer. Assoc. Cancer Res.*, **30**, 120.

LAIDLAW, I., POTTEN, C.S., CLARKE, R. & HOWELL, A. (1992). The effect of oestrogen and progesterone on the proliferation of normal human breast tissue xenografts in nude mice (manuscript in preparation).

MARGISON, G.P. & O'CONNOR, P.J. (1990). Biological consequences of reaction with DNA: role of specific lesions. In *Chemical Carcinogenesis and Mutagenesis*, Cooper, C.S. & Grover, P.L (eds) Handbook Exp. Pharmacology. 94/I Springer-Verlag, Berlin, Heidelberg, pp 547-571.

MCMANUS, M.J. & WELSCH, C.W. (1984). The effect of estrogen, progesterone, thyroxine and human placental lactogen on DNA synthesis of human breast ductal epithelium maintained in athymic nude mice. *Cancer*, **54**, 1920-1927.

MEYER, W.H., LOFFIN, S.K., HOUGHTON, J.A. & HOUGHTON, P.J. (1990). Accumulation, intracellular metabolism and antitumour activity of high- and low-dose mexthotrexate in human oesteosarcoma xenografts. *Cancer Commun.*, **2**, 219-229.

O'CONNOR, P.J., FIDA, S., FAN, C.Y., BROMLEY, M. & SAFFHILL, R. (1988). Phenobarbital: a non-genotoxic agent which induces the repair of $O^6$-methylguanine from hepatic DNA. *Carcinogenesis*, **9**, 2033-2038.

O'CONNOR, P.J., FAN, C.Y., ZAIDI, S.N.H. & COOPER, D.P. (1991). Selective alkylation of cells in rat tissues after treatment with N-nitrosocompounds: immunohistochemical detection of potential target cells. In *Biomonitoring and Carcinogen Risk Assessment*, Garner, R.C., Farmer, P.B., Steel, G. & Wright, A.S. (eds), Oxford University Press, pp 355-362.

PITOT, H.C. (1990). Mechanisms of chemical carcinogenesis: theoretical and experimental bases. In *Chemical Carcinogenesis and Mutagenesis*, Cooper, C.S. & Grover, P.L. (eds). Handbook Exp. Pharmacology **94/I**, Springer-Verlag, Berlin, Heidelberg, pp 3-29.

POTTEN, C.S., WATSON, R.J., WILLIAMS, G.T., TICKLE, S., ROBERTS, S.A., HARRIS, M. & HOWELL, A. (1988). The effect of age and menstrual cycle upon proliferative activity of the normal breast. *Br. J. Cancer*, **58**, 163-170.

ROFSTAD, E.K. (1990). PLD - repair in human melanoma xenografts following single dose and fractionated irradiation. *Br. J. Cancer*, **61**, 856-860.

SAFFHILL, R., FIDA, S., BROMLEY, M. & O'CONNOR, P.J. (1988). Promutagenic alkyl lesions are induced in the tissue DNA of animals treated with isoniazid. *Human Toxicol.*, **7**, 311-317.

SAFFHILL, R., MARGISON, G.P. & O'CONNOR, P.J. (1985). Mechanisms of carcinogenesis induced by alkylating agents. *Biochem. Biophys. Acta.* (Cancer Reviews), **823**, 111-145.

SCHOLD, S.C., BRENT, T.P., VON HOFFE, E., FRIEDMAN, H.S., MITRA, S., BIGNER, D.D., SWENBERT, J.A. & KLEIHUES, P. (1989). $O^6$-Alkylguanine-DNA alkyltransferase and sensitivity to procarbazine in human brain tumor xenografts. *J. Neurosurg*, **70**, 573-577.

WILD, C.P., SMART, G., SAFFHILL, R. & BOYLE, J.M. (1983). Radioimmunoassay of $O^6$-methylguanine in DNA of cells alkylated *in vitro* and *in vivo*. *Carcinogenesis*, **4**, 1605-1609.

WILD, C.P., MONTESANO, R., VAN BENTHEM, J., SCHERER, E. & DEN ENGELSE, L. (1990). Intercellular variation in levels of adducts of aflatoxin $B_1$ and $G_1$ in DNA from rat tissues: a quantitative immunocytochemical study. *J. Can. Res. Clin. Oncol.*, **116**, 134-140.

YOHN, M.D., LEHMAN, T.A., KURIAN, P., RIBOVICH, M. & MILO, G.E. (1988). Benzo(a)pryrene diol epoxide I modification of DNA in human skin xenografts. *J. Invest. Dermatol.*, **91**, 363-368.

ZAIDI, S.N.H. & O'CONNOR, P.J. Comparison of N-nitrosodimethylamine induced DNA methylation at tissue and cell specific levels and basal levels of $O^6$-alkylguanine-DNA alkyltransferase in the female, athymic nude and BDF, mouse (in preparation).